# Reverse genetics in Chlamydomonas: a platform for isolating insertional mutants

**DOI:** 10.1186/1746-4811-7-24

**Published:** 2011-07-27

**Authors:** David Gonzalez-Ballester, Wirulda Pootakham, Florence Mus, Wenqiang Yang, Claudia Catalanotti, Leonardo Magneschi, Amaury de Montaigu, Jose J Higuera, Matthew Prior, Aurora Galván, Emilio Fernandez, Arthur R Grossman

**Affiliations:** 1 Department of Plant Biology, The Carnegie Institution for Science, Stanford, CA 94305, USA; 2 Departamento de Bioquímica y Biología Molecular, Universidad de Córdoba, Córdoba 14071, Spain; 3 National Center for Genetic Engineering and Biotechnology (BIOTEC), National Science and Technology Development Agency, Pathumthani, 12120, Thailand; 4 Montana State University, Department of Chemical and Biological Engineering, and Department of Microbiology, Bozeman, MT 59171, USA; 5 PlantLab, Scuola Superiore Sant'Anna, 56127 Pisa, Italy; 6 Max Planck Insitute for Plant Breeding Research, Department of Plant Developmental Biology, D-50829, Köln, Germany

**Keywords:** reverse genetics, insertional mutants, transformation, mutant library, mutant screening, paromomycin resistance, PCR-based screening

## Abstract

A method was developed to identify insertional mutants of *Chlamydomonas reinhardtii *disrupted for selected target genes. The approach relies on the generation of thousands of transformants followed by PCR-based screenings that allow for identification of strains harboring the introduced marker gene within specific genes of interest. Our results highlight the strengths and limitations of two independent screens that differed in the nature of the marker DNA used (PCR-amplified fragment containing the plasmid-free marker versus entire linearized plasmid with the marker) and in the strategies used to maintain and store transformants.

## Background

Forward genetics screens to isolate insertional mutants with specific phenotypes have been used successfully to identify genes involved in different metabolic and regulatory pathways in the eukaryotic green alga *Chlamydomonas reinhardtii *(Chlamydomonas throughout) [1-3]. However, these screens rely on selectable phenotype-based assays, making it impossible to identify lesions in genes that do not result in a clearly measurable phenotype. Recently, the sequencing of the entire Chlamydomonas nuclear genome [4] has revealed many putative genes encoding proteins with no known biochemical functions. To dissect the functions of such proteins, as well as the functions of individual members of multi-protein families, a robust approach for targeting mutations in specific gene is required. In contrast to forward genetics, reverse genetics approaches target specific gene mutations; the most facile methods for targeting gene lesions can be developed for organisms in which the nuclear DNA can be manipulated through homologous recombination [5,6]. However, homologous recombination does not occur at a high frequency, relative to non-homologous recombination, in most eukaryotic organism, including plants and algae.

Within the last decade, some alternative approaches have been developed to generate specific mutations in organisms in which homologous recombination is a low frequency event. In Arabidopsis and rice, transformation with *Agrobacterium tumefaciens *T-DNA has led to the generation of hundreds of thousands of transformants with T-DNA insertions distributed with low bias in the genome [7,8]. Defining the insertion site for each transformant has allowed for the establishment of sequence-indexed libraries of mutant plants that can be stored as seed at low cost and for long periods of time. The availability of these libraries affords the scientific community the opportunity to characterize Arabidopsis and rice lines with a lesion in nearly any gene [7,8]. An indexed insertional mutant library in which the inserts are precisely located has not been established for Chlamydomonas, primarily because maintenance of a large collection of mutants of this alga is difficult. Chlamydomonas cultures are most commonly maintained as vegetative cells on agar-containing medium for short periods of time; these cultures need to be refreshed every few months and the quality of the mutant population will likely deteriorate over time. Long-term storage of Chlamydomonas cultures can be achieved by cryopreservation under liquid nitrogen [2,9], although this method is time consuming, has low recovery rates and high costs. These features make the establishment and maintenance of a permanent mutant collection for Chlamydomonas inconvenient.

The recent use of zinc-finger nucleases represents an elegant approach to promote site-directed mutations in plants [10-13]. This approach employs custom-designed chimeric endonucleases that can specifically cleave target genes and promote, through naturally-occurring non-homologous recombination processes, the occurrence of small deletions and/or insertions within the gene of interest. The successful generation of specific mutants can be achieved with a moderately small population of transformants since for each transformant line, 2-16% of the transformants contain lesions in the target gene. However, the approach is both time consuming and labor intensive, requiring both the design and construction of chimeric endonucleases; these features of the technique reduce its utility as a high-throughput means for generating specific mutants. Moreover, the relatively recent development of this technology is reflected in the limited collection of zinc-finger nucleases and genomic target sites that can be used in different target organisms. Although some *in silico *analyses of putative zinc-finger nucleases and genomic target sites for Chlamydomonas have been identified [14], to our knowledge there is no report that demonstrates the successful use of this approach for generating mutants in this alga.

Another approach for generating lesions in specific genes is TILLING (Targeting Induced Local Lesions In Genomes). TILLING is a relatively high-throughput reverse genetics strategy that has been used in plants to generate allelic series of chemically-induced point mutations in genes of interest. While researchers are applying this technique to Chlamydomonas http://www.chlamy.org/abstracts2010/niyogi.htm, its utility for identifying specific mutants of this alga has not been documented. TILLING requires a small population of mutants relative to insertional mutagenesis since each individual can carry multiple lesions (up to 1 mutation per kb). However, in addition to the relatively high cost of this technique, it has some disadvantages [15]. Many lesions within the target gene will either not impact the activity of the encoded protein or cause partial loss of activity that could result in a wide range of phenotypic strengths. Moreover, since each individual harbors multiple mutations, backcrossing and segregation analysis must be performed to eliminate unwanted background lesions. Finally, multiple mutant alleles of the same target gene are required to demonstrate linkage between the phenotype and the lesions, which can make the overall process extremely time consuming.

Another reverse genetic approach involves RNA silencing; this strategy has been successfully used to suppress endogenous transcript levels in Chlamydomonas [16-18]. Similar to the zinc nuclease strategy, RNA silencing does not require the generation of a large mutant population although it does involve labor intensive DNA constructions and molecular analyses of several transgenic lines to determine target gene expression levels. Moreover, it is difficult to obtain a complete loss-of-function RNA-silenced strain and there may be off-target effects of the introduced construct [15].

Finally, reverse genetics approaches based on the screening of insertion libraries by PCR have been used to identify specific mutants in a variety of plants [19-22]. The screening of an insertional library through a reverse genetics approach has been used before with Chlamydomonas [23], although in this case the authors used a hybridization-based method to screen a subpopulation of phenotypically pre-selected transformants; phenotype pre-selection of transformants and laborious hybridization-based procedures make this approach impractical for high throughput analysis. This manuscript describes the optimization of PCR-based screenings of insertional libraries in order to establish a platform for identifying specific Chlamydomonas mutants. Our results demonstrate that this methodology can be used as an efficient procedure for isolating transformants disrupted in specific genes. The steps required for execution of this procedure are discussed.

## Results and Discussion

### A. General overview

**Figure 1:**
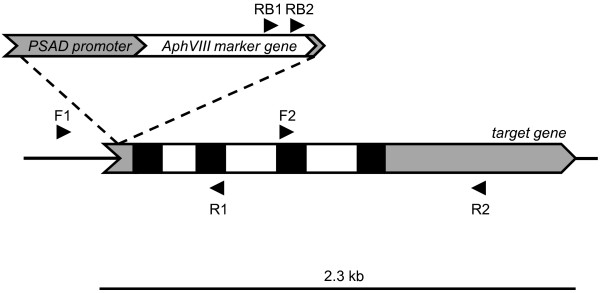
**Primer design**. Depicted is an actual case of the Chlamydomonas RDP3 target gene (ID, 183511) interrupted by the marker gene. The marker gene consisted of a PCR-amplified fragment containing the *AphVIII *gene under the control of the PSAD promoter, and with an incomplete 3'UTR. Since the DNA marker can be integrated into the Chlamydomonas genome in two different orientations, two forward (F1 and F2) and two reverse target gene primers (R1 and R2) were used to identify the insertion site. Typically, forward and reverse primers are separated by ~1.0 kb along the sequence of the target gene. Each target gene primer is used in combination with individual marker gene primers (RB1 and RB2) in independent PCR amplifications. Amplification would only be possible in those transformants in which the marker gene is inserted close to or within the target gene. In the example depicted, the marker gene was inserted within the 5'UTR of the target gene and amplification was observed with the RB2-R1 primer pair. Exons, introns and UTR regions are represented by black, white and grey squares respectively.

**Figure 2:**
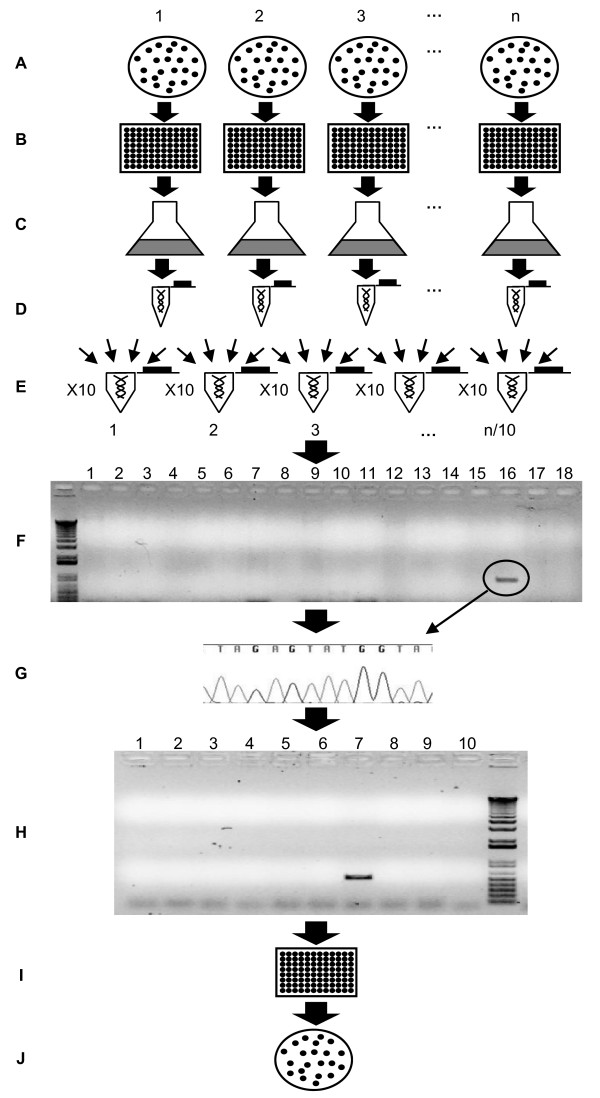
**Work-flow diagram used for the reverse genetics approach**. ***A**. Generation and selection of transformants*. Thousands of transformants are generated using the *AphVIII *marker gene and selected on paromomycin-containing plates. ***B**. Isolation of individual transformants*. Individual colonies are cultured in 96-well microtiter plates (200 μl per well). ***C**. Transformant pooling*. Aliquots (25-50 μl) from each well of an individual 96 well microtiter plate are pooled and cultured in fresh medium (pool). ***D**. Isolation of genomic DNA from pools*. DNA from each pool of transformants is isolated and diluted to 100 ng/μL. ***E**. Generation of DNA superpools*. Superpools are constructed by combining equal volumes of genomic DNA from 10 different pools. ***F**. Screening the superpools*. A set of independent PCR reactions using marker and target gene primers is performed with each DNA superpool as template (in the real case depicted, amplification occurred in the superpool sample loaded in lane 16) ***G**. Confirmation of PCR products*. Amplified PCR products are sequenced using the marker gene primer. ***H**. Screening specific pools*. The 10 different pools of DNA that comprise the superpool are individually screened using the appropriate primer pair. ***I**. Screening of individual transformants*. Positive transformants are identified within a specific microtiter plate by colony PCR. ***J**. Isolation of specific transformants*. Cells from the well containing the positive transformant are streaked onto solid medium to obtain single cell-derived colonies, which are then screened by colony PCR. This step is used to eliminate potential cross-contamination among transformants.

A PCR-based reverse genetics approach was developed to isolate strains with lesions in specific Chlamydomonas target genes from libraries of transformants in which the *AphVIII *marker gene, which encodes the aminoglycoside 3'-phosphotransferase from *Streptomyces rimosus *and confers resistance to the antibiotic paromomycin [24], was inserted randomly into the nuclear genome. The overall strategy behind this approach is to generate individual pools of genomic DNA from thousands of different transformants that have been previously indexed in 96-well microtiter plates. These pools of genomic DNA are then screened using PCR reactions in which one of the primers anneals to the marker gene and the other anneals along the target gene sequence. In some instances, the target gene primers would be located close enough to the marker gene insertion to allow amplification, which in turn would lead to the identification of specific strains in which the marker gene had inserted into the target gene, potentially disrupting its function. We have used this approach to screen two independent transformant libraries: one of the libraries, described as Library 1 below, contained ~100,000 transformants, while a second library, described as Library 2, was generated earlier [2] and contained ~22,000 transformants. The main features of these transformant libraries are presented in Table 1. The procedures used to construct Library 1 and the work flow for the PCR-based screening are described in the following sections and summarized in Figures 1 and 2.

**Table 1:** Main features of transformant libraries used for identification of strains harboring specific gene disruptions

	Number of transformants generated	Marker used for transformation	Transformation method	Indexing method	Preservation method	Reference
**Library 1**	~100,000	*AphVIII* (1.7 kb PCR-fragment)	electroporation	individuals	none (one-use library)	[35]; this work
**Library 2**	~22,000	*AphVIII* (4.7 kb linearized plasmid)	glass beads	pools of 96	cryopreservation	[2]

### B. Marker gene DNA used for insertional mutagenesis

**Figure 3:**
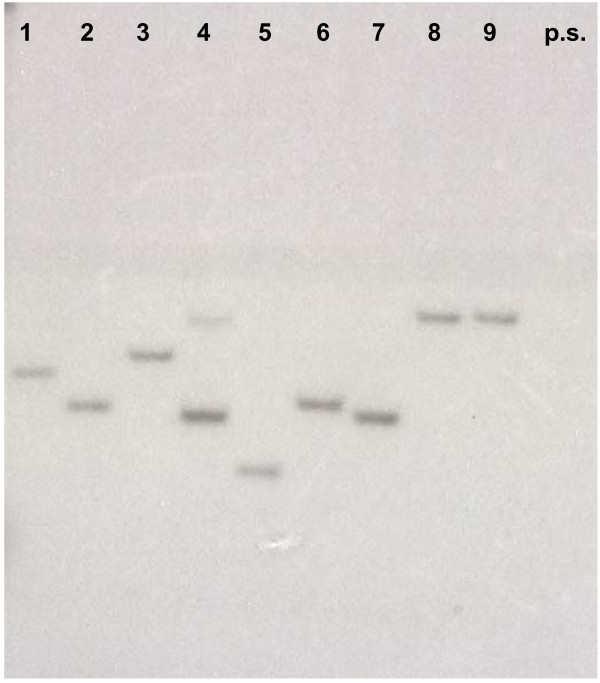
**Determination of marker gene copy number by Southern blot hybridization**. Southern-blot analysis of digested (*PstI*) genomic DNA from 9 randomly isolated paromomycin resistant mutants. A fragment of the *AphVIII *gene labeled with ^32^P-dNTPs was used as probe. As shown, most transformants have a single copy of the integrated marker gene. Only the transformant represented in lane 4 may have two insertions (indicated by the two hybridizing bands). p.s., parental strain used to obtain the transformants.

Although a number of different marker genes have been used for developing transformation of Chlamydomonas [25], we chose the *AphVIII *gene, which confers paromomycin resistance to the organism [24]. Our preference is based on the stability of the drug-resistance phenotype of transformants and the fact that expression of this gene in algal cells does not appear to elicit the generation of random, spontaneous mutations. Moreover, the *AphVIII *is a heterologous gene, making it easy to design primers specific for the *AphVIII *sequence, which limits nonspecific amplification events. Previously, a linearized plasmid containing the *AphVIII *gene under the control of the *RBCS-HSP70 *chimeric promoter and the *RBCS *terminator was used to generate ~22,000 Chlamydomonas transformants (Table 1) [2]. In a second configuration, the marker DNA was a 1.7 kb plasmid-free, PCR-amplified DNA fragment containing the *AphVIII *gene under the control of *PSAD *promoter [26], and without a terminator sequence. PSAD is a nuclear-encoded chloroplast protein required for photosynthetic electron transport. The use of plasmid-free DNA markers obtained by PCR amplification or enzymatic digestion has specific advantages when used for insertional mutagenesis in reverse genetic screens. With insertion of a plasmid-free marker into the genomic DNA, the distance from the end of the selectable sequence to the beginning of both genomic flanking regions of the target gene can be very short. In contrast, when an entire circular or linearized plasmid marker DNA is used for transformation, this distance may be long, which makes it more difficult to identify interrupted target genes. Moreover, there are often deletions and DNA rearrangements in the vicinity of the junctions between the marker and genomic DNA, making it difficult to define the exact sequence at which the exogenous marker gene is integrated; this is especially problematic for the nonselectable regions of the plasmid where there is less constraint on the arrangement of the sequence. Hence, DNA fragments containing only the marker genes without plasmid sequences facilitate the design of reliable marker-specific primers situated very close to genomic flanking regions, which is an important advantage for identifying the interrupted genes. Furthermore, we have observed in some instances that the transformation procedure caused integration of short plasmid sequences and incomplete DNA markers, which could interfere with gene functions but would not be linked to the marker gene. The use of short plasmid-free markers likely reduces the risk of obtaining insertional mutations caused by non-marker- or incomplete marker-associated insertions that could complicate analysis of the mutants generated. Table 2 summarizes the benefits of using a plasmid-free marker for transformation relative to the entire plasmid harboring a marker gene. Finally, it is important to consider the concentration of marker gene DNA used for each transformation; this concentration will impact the number of transformants obtained per transformation event and the number of integrated marker gene copies per transformant. The ideal situation would be the generation of high numbers of individual transformants with a single copy of the marker gene DNA integrated into the genome; this would simplify linkage analysis (testing whether the phenotype of the strain is linked to the insertion). We used 100 ng of PCR amplified marker gene DNA per transformation in Library 1, which generated 100-500 transformants per electroporation event with an average of 1.1 copies of the insert per transformant (Figure 3), suggesting that 90% of the transformants harbored a single copy of the integrated marker DNA. Similarly, 100 ng of linearized whole plasmid containing the *AphVIII *marker gene was used to generate Library 2 and the number of insertions per transformant was also close to one [2].

**Table 2:** Benefits of the use of plasmid-free markers relative to an entire plasmid harboring a marker gene when performing reverse genetic screens.

	Distance from the inserted marker gene to the host genomic DNA	Stability of the maker primer binding sites on the integrated marker DNA	Risk of non-selectable insertions (partial plasmid sequences or incomplete marker genes)
**Plasmid-free marker DNA (Library 1)**	Short (as short as few bp) for both borders of the marker DNA	Stable if placed within the marker gene coding sequence	Low^1^
**Linearized entire plasmid marker DNA (Library 2)**	Long (up to few kb) for at least one border of the marker DNA	Can be lost if placed close to the borders of the linearized vector	High^2^

^1^Rearrangement in, or fragmentation of the introduced marker would eliminate the drug resistance selection; no transformant would be identified. ^2^Rearrangements and fragmentation could occur that would leave the marker gene intact, which would allow fragments of non-selectable DNA to integrate into the genome and cause secondary mutations.

### C. Primer design

#### (i) Target primers

We designed a set of specific primers for each target gene; each set contained forward and reverse primers. Both forward and reverse primers were used because the heterologous integration of the marker DNA into the Chlamydomonas genome can occur in two different orientations. The primers in each direction were separated by ~1.0 kb (Figure 1). For a positive transformant, a spacing of 1 kb between target gene primers would result in a PCR product no larger than 1 kb, which would yield a high probability of detecting a positive signal. Decreasing the distance between the primers would increase the chances of detecting an insertion in the target gene, although it would make the procedure more costly and time consuming since a larger number of PCR reactions must be performed (see section G). Also, it is possible to design primers upstream and downstream of the target gene to capture mutants in which the target gene undergoes either complete or partial deletion during the transformation process. In order to use a single protocol for all PCRs, the primers were designed to have similar Tm values (~60ºC +/- 3°C). Target primers with potential false annealing binding sites within the target sequence were rejected. Moreover, target primers predicted to anneal to highly repetitive or conserved regions of target genes were also usually rejected.

#### (ii) Marker gene primers

RB1 and RB2 primers were used as *AphVIII*-specific primers (Figure 1). Both are forward primers situated in the 3' region of the *AphVIII *coding sequence. The best results were obtained with the RB2 primer (lower number of non-specific amplifications), and this primer was used almost exclusively for the initial screening of the library; the RB1 primer was frequently used to confirm positive amplification reactions. It would also be possible to use reverse primers situated in the 5'region of the marker gene, although such primers were not used in this work. Furthermore, using marker gene primers within coding sequences (CDS) is recommended (rather than generating primers within promoters or terminator regions of the markers) since marker genes are usually heterologous genes and marker gene primer binding sites are less likely to be present in the genome of the host organism. In contrast, the promoter and terminator regions used to drive expression of marker genes are often derived from endogenous Chlamydomonas sequences [25], making it impossible to design specific primers to these sequences. The use of primers that anneal to the coding region of the marker gene also increases the probability of detecting selectable insertions containing the entire marker gene and stable primer binding sites; as previously mentioned, sequences at marker DNA-Chlamydomonas genomic DNA junctions can undergo rearrangements and deletions as a consequence of recombination, and consequently primer binding sites near to the border sequences may be lost.

#### (iii) Primer optimization and negative controls

Before initiating PCR screening of the genomic DNA from transformants, it is highly recommended that controls be performed with genomic DNA isolated from the parental strain (in this case strain D66). These controls involve performing PCR with each target primer individually (asymmetric PCR) and also in combination with the RB2 and RB1 marker gene primers. A target primer that alone or in combination with the RB2/RB1 primer yields an amplification product from the parental strain genomic DNA should not be used for the screens. We also tested various target primer cocktails in combination with the marker gene primers. The primer cocktails contained mixtures of different forward or reverse primers (at equimolar concentrations). Identifying primer cocktails that yield no amplification products from the parental strain genomic DNA reduces the number of PCR reactions needed for a successful screening of the transformant libraries (see section G).

### D. Isolating individual transformants

Under optimal conditions, we obtained an average of 100-500 colonies per transformation. Individual colonies were picked by touching them with the tip of a toothpick, and transferring them to a well of a 96-well microtiter plate. Each of the 96-well microtiter plates is considered a "pool". To generate an unbiased library that was not impacted by the growth rate of individual transformants (some transformants grew much more slowly than others), independent colonies were picked once their sizes were clearly visible on the plate, which often required a different number of days for growth.

### E. Maintenance of transformants

The most commonly used method for storing Chlamydomonas cells involves allowing them to grow on solid agar medium at low light and then transferring them to new medium every few months. An alternative to this method is cryopreservation in liquid nitrogen [2,9]. Preparing the individual transformants for cryopreservation is time consuming and the frequency of recovery following the thawing process is low; between 3-6% of the initial cell population is recovered [2]. Furthermore, some mutant strains may be much more sensitive to the cryopreservation procedure than others. Since at this point there is no highly reliable, straightforward method for long-term storage of Chlamydomonas cells, the maintenance of thousands of individual clones is logistically complicated. The maintenance method selected for storage of Library 2 was cryopreservation of the pooled strains from each of the 96-well microtiter plates; no individual transformants were maintained [2]. For the more recent screen (Library 1), we decided to generate a 'one-use' library; individual transformants were maintained for just the time needed to perform the PCR screenings and the library was discarded after individual positive transformants were isolated. If necessary, transformants were kept viable for an additional period (1-2 months) by transferring small aliquots from each well to new 96-well microtiter plates.

### F. Isolating DNA and constructing DNA pools and superpools

Aliquots of cells from each well of an individual 96-well plate were combined and used to inoculate a 'transformant pool culture'. The transformant pool cultures were then used for genomic DNA isolation; the DNA preparation from each pool culture represents a DNA "pool". For Library 1, 1041 independent pool cultures were obtained. The DNA prepared from each pool was diluted to 100 ng/μL and equal volumes of 10 different DNA pools were combined to generate a DNA "superpool". Hence each DNA superpool contains the genomes of 960 individual transformants. We performed experiments to determine the optimal superpool size. A pool containing a characterized transformant with the *AphVIII *marker gene inserted into a specific target gene was used as a positive control. Dilution of the genomic DNA from this strain with DNA from other pools provided information on how large a dilution could be tolerated before detection of a specific insertion within the superpool DNA was severely compromised. The results indicated that a combination of equal amounts of DNA from 10 different pools resulted in the largest dilution that still allowed reproducible amplification of the positive control (data not shown). Hence, we combined 10 different pools to a final concentration of 100 ng/μL of total genomic DNA to yield 104 independent 'superpools' for Library 1.

### G. PCR screening of superpools and sequencing of PCR products

DNA superpools were used as a template for the PCR-based screen. For each superpool several independent PCR reactions were performed using the RB2 primer in combination with different target gene primers. The approximate number of PCR reactions (*N*) required to screen several target genes in a library depends on the total number of transformant isolated (*Tn*) and the number of target gene primers used (*TPn*). If DNA superpools from 960 transformants are used *N *can be defined as *N *= (*Tn *÷ *960*) × *TPn*. Where *TPn = ((∑ Kb target genes sequences) × 2) *when target gene primer are separated by 1 kb and both forward and reverse target gene primers are used. PCR products were resolved on agarose gels, excised from the gels, purified, and sequenced using the RB2 primer; sequencing was used to confirm positive PCR products in the superpools. If necessary, low abundance amplification products were re-amplified prior to sequencing, as previously described [27].

### H. Screening pools and isolating the positive transformants

Once a specific positive PCR product is detected in a superpool, the 10 DNA pools within that superpool are individually screened by PCR (using the same conditions and primers that were used to obtain the positive PCR product from the superpool). PCR products from individual pools were sequenced (as described above) to confirm their identities. Further characterizations of the pools, as described below, enabled identification of the individual strains harboring insertions of interest. As Libraries 1 and 2 were maintained differently (transformants in Library 1 were maintained as individual colonies whereas transformants in Library 2 were maintained as pooled cultures), two different approaches were employed to identify a positive transformant within a pool. For Library 1, we combined transformants of individual rows and columns of each 96-well microtiter plate (10 μL of each transformant) and assayed the pooled samples for a given row and column by colony PCR. This allows for identification of the row and column in which a transformant of interest occurs, with the combined row and column information pinpointing the exact position of the transformant in the microtiter plate. An aliquot is then taken from the well containing the positive transformant and plated onto solid agar medium to obtain individual colonies. A few of these individual colonies are tested by colony PCR to identify single transformants with the desired gene disruption. This last step is required to eliminate potential cross contamination with transformants from other wells of the same microtiter plate; cross contamination among wells is not uncommon when there is significant manipulation of plates. Because transformants of interest in Library 2 were only maintained in a culture with 95 other strains, cultures containing the desired transformants were serially diluted and plated onto solid agar medium to obtain individual colonies. PCR was performed on randomly-chosen individual colonies to identify those transformants harboring insertions in the target genes. The maintenance of transformants as a pooled culture has clear disadvantages when trying to isolate a specific transformant within the pool; it can be very time consuming, especially if transformants of interest grow slowly and are under-represented within the population.

### I. Reliability of the procedure and insertion features

With respect to the 63 target genes screened for in Library 1, 64 independent insertions were identified that represented disruptions of 52 of the different target genes (Table 3); for some genes more than one independent insertion was identified. Of the 64 independent insertions identified, we isolated 45 of the corresponding transformants, which had disruptions in 37 different target genes (Table 3). For 19 independent insertions the corresponding transformants could not be isolated because they died during the maintenance period; 7 of these independent insertions were associated with 3 different genes. It is also possible that mutations in these genes compromised the viability of the cells.

**Table 3:** Number of insertions identified and transformants isolated in Libraries 1 and 2.

		**Insertions identified**		**Transformants isolated**	
	**Screened genes**	**total**	**in different genes**	**total**	**in different genes**
	
**Library 1**	63	64	52	45	37
**Library 2**	7	4	4	2	2

For the experiment with Library 1, there was an 82.5% success rate for identifying insertions within the screened genes and a 58.7% success rate for isolating the corresponding transformants. Naturally, this success rate will dependent upon the size of the library being screened, the size of each target gene, and the nature of the gene products (the loss of some proteins will severely compromise cell fitness). Although Library 1 contained ~100,000 transformants, we did not generate all of the transformants at the same time; two rounds of transformations were performed at two different times and the population of transformants generated at each time was designated a 'Sub-library'. For each Sub-library, roughly 50,000 transformants were arrayed. Furthermore, we did not screen the entire population of each Sub-library for all of the target genes but used a progressive screen for individual target genes; the screen for each target gene was often terminated once an appropriate insertion was identified. In our case, some interrupted target genes were identified in the first 3,000 transformants assayed.

Library 2 was used to identify insertions in 7 genes (Table 3). From this screen there was a 57.1% success rate for identifying insertions in specific target genes, which is significantly lower than the level of success with Library 1 (82.5%). This difference is likely due to the low number of total transformants in Library 2 relative to Library 1 (22,000 compared to 100,000 transformants) and to the existence of large deletions in Library 2 (see below) that may decrease the probabilities of generating PCR products for specific insertions; deletions in the target genes will eliminate target primer binding sites. Moreover, transformants ultimately isolated as single colonies (2) represented 50% of the identified insertions (4) for Library 2 (Table 3). As stated previously, isolating specific transformants by random colony PCR analysis from cultures representing a pool of 96 different transformants can be time consuming and difficult, especially if the transformant of interest grows slowly or is under-represented within the population. Furthermore, a loss of cell viability as a consequence of thawing/defrosting processes may also impact the identification of specific transformants. However, the low number of screened genes (7) and insertions identified (4) in Library 2 does not provide us with statistically relevant information and additional screenings would have to be performed before we can be sure of the major impacts of the Library 2 screening procedure on mutant isolation

It is noteworthy that mutants generated by electroporation using the *PSAD*:*AphVIII *PCR-fragment (Library 1) had no or very small deletions (0-34 bp) of the genomic region at the site of marker gene insertion (Tables 4 and 5). In contrast, transformants in which the *AphVIII*-containing whole plasmid was introduced into cells by the glass bead method of transformation (Library 2) often contained large deletions at the insertion site (up to 35 kb). Differences in the frequency and sizes of the deletions that occur during the integration of the marker gene in Libraries 1 and 2 (Table 4) should be considered since such differences could impact the phenotypes of the various transformants and the gene coverage of the libraries; libraries containing transformants with large deletions would require a smaller population of individual transformants to saturate the genome (e.g. to generate a population having at least one insertion in every gene) [23]. Differences in deletion size probably reflect the type of marker used (a 1.7 kb PCR-product was used to construct Library 1 while a 4.7 kb plasmid was used to construct Library 2) and/or the method of transformation (electroporation for Library 1 and glass beads agitation for Library 2); most likely both influence features at the site of marker gene insertion.

**Table 4:** Sizes of genomic deletions generated by insertion of the marker gene.

	List of deletions sizes* (bp)	**n**
	
**Library 1**	0 (×23), 2, 3, 4, 5 (×2), 8, 12 15, 34	32
**Library 2**	0 (×3), 12, 22, 200, 4178, 5000, 10000, 12000, 35000	11

**n**, number of transformants analyzed. **x**, the number of times that the specified deletion size was found. 0 denotes that the insertion was 'true' with no deletion at either flank. *some deletion size analyses were noted for transformants isolated by forward genetic screens not described in this manuscript [36-38].

**Table 5:** Isolated transformants.

Interrupted gene	ID^1^	Library	Localization and orientation of the insertion^2^	Deletion size at the genomic region (bp)	Expression of the interrupted gene
*Sulfur starvation responsive genes*					
*RDP3*	183511	1	5'UTR, +	n.a.	n.a.
*AOT4*	206110	1	1^st ^exon, +	0	truncated and co-expressed with the marker as a chimeric mRNA
*TAUD1*	127464	1	2^nd ^intron, +	0	truncated and co-expressed with the marker as a chimeric mRNA. Low expression levels relative to the parental strain
*TAUD2*	77600	1	3^rd ^exon, -	n.a.	mRNA not detected
*HAP3*	182794	1	4^th ^exon,+	n.a.	n.a.
*PWR1*	205514	1	1^st ^exon, +	0	truncated and co-expressed with the marker as a chimeric mRNA.
*Sulfate transporter genes*					
*SULTR1 *(1)	420357	1	3'UTR, +	8	n.a.
*SULTR1 *(2)	420357	1	promoter, +	3	n.a.
*SULTR1 *(3)	420357	1	17^th ^exon, +	0	truncated and co-expressed with the marker as a chimeric mRNA
*SULTR2*	150514	1	9^th ^exon, -	0	truncated and co-expressed with the marker as chimeric mRNA; transcript has a premature stop codon after splicing
*SLT1 *(1)	205502	1	8^th ^exon, +	0	truncated and co-expressed with the marker as chimeric mRNA
*SLT1 *(2)	205502	2	promoter, +	12,000	n.a.
*SLT2*	205501	1	5'UTR, +	0	low expression levels relative to the parental strain
*Hydrogen production and fermentation related genes*					
*PFL1 *(1)	206677	1	7^th ^exon, +	0	truncated and co-expressed with the marker as a chimeric mRNA
*PFL1 *(2)	206677	1	1^st ^intron, -	12	low expression levels relative to the parental strain
*PFL1*(3)	206677	1	5'UTR, -	0	low expression levels relative to the parental strain
*FMR*	145357	1	5'UTR, +	n.a.	n.a.
*MME4*	196831	1	6^th ^exon, +	0	n.a.
*HYDA1 *(1)	183963	1	5'UTR, +	n.a.	n.a.
*HYDA1 *(2)	183963	1	3'UTR, -	2	n.a.
*HYDA2*	24189	1	9^th ^exon, +	0	n.a.
*PDC1*	193810	1	5^th ^introns, -	n.a	n.a
*SEP *(1)	147682	1	4^th ^exon, +	0	n.a.
*SEP *(2)	147682	1	7^th ^exon, +	4	n.a.
*NADTH *(1)	139758	1	3'UTR, -	0	n.a.
*NADTH *(2)	139758	1	3'UTR, -	n.a.	n.a.
*PDK1*	196270	1	3^rd ^intron, +	0	n.a.
*H4*	163170	1	5'UTR, -	n.a.	n.a.
*FDX*	188740	1	2^nd ^exon, +	15	n.a.
*P4H-2*	206683	1	3^rd ^exon, +	0	n.a.
*P4H-10*	111255	1	7^th ^exon, +	7	n.a.
*P4H-10b*	182877	1	3'UTR, -	0	n.a.
*HCP4*	148255	1	3'UTR, -	34	n.a.
*ADH1*	536345	1	15^th ^exon, -	0	no protein detected
*Other processes*					
*RelA/SpoT *homolog (1)	419232	1	promoter, +	0	n.a.
*RelA/SpoT *homolog (2)	419232	1	promoter, +	0	n.a.
*AMT1;1*	158745	2	3'UTR, -	n.a.	n.a.
*CLPD*	195417	1	3'UTR, +	n.a.	n.a.
*PK2*	174928	1	16^th ^exon, +	n.a.	n.a.
*WD*	524057	1	17^th ^exon, +	0	n.a.
*GIP1*	34332	1	2^nd ^exon, +	0	n.a.
*ABC1*	523148	1	3'UTR, +	n.a.	n.a.
*ABC2*	60710	1	23^rd ^exon, +	n.a.	n.a.
*GOX8*	196818	1	2^nd ^exon, -	n.a.	n.a.
*GOX15*	196829	1	16^th ^exon, +	0	n.a.
*ATPASE*	190023	1	3'UTR, -	1	n.a.
*KIN*	140337	1	5'UTR, +	n.a.	n.a.

^1^Protein IDs from the Joint Genome Institute Chlamydomonas v.4 website. ^2^Localization and orientation of the inserted marker gene relative to the interrupted target gene: + and - denote the same and opposite orientation, respectively. Independent insertion alleles for the same target gene are indicated by the same gene designation, but with a different number placed in a parenthesis. n.a.: not analyzed.

Analysis of both libraries used in this study suggested that the inserted DNA did not exhibit preferential sites of integration within genes; the inserts were randomly distributed along the genes (exon, introns, UTRs and promoters) (Table 5). However, for Library 1 we did observe that the majority of *AphVIII *gene insertions were in the same orientation as the interrupted gene; out of 48 transformants analyzed 34 had the marker inserted in the same orientation as the target gene (70.8%). Moreover, in the case of Library 1, some transformants were identified in which the interrupted gene was co-transcribed with the *AphVIII *marker gene. We attribute these two findings (bias in orientation of the *AphVIII *cassette and co-transcription of interrupted gene with *AphVIII*) to the absence of a terminator sequence in the *AphVIII *cassette that was used for transformation; transcriptional read-through from the *AphVIII *promoter into the interrupted Chlamydomonas gene could occur, which may confer stability to the *AphVIII *mRNA. These chimeric mRNAs are unlikely to serve as a template for active synthesis of the protein encoded by the disrupted gene (the proteins generated would often be truncated and/or be encoded by a different reading frame of the mRNA). This system could be improved by inclusion of a terminator sequence at the 3' end of the *AphVIII *marker gene. Finally, based on qPCR results, the mRNAs from the disrupted genes were usually either absent of accumulated to reduced levels relative to that of the parental strain (data not shown).

## Conclusions

The generation of insertional transformant libraries of Chlamydomonas and the subsequent PCR-based screening of those libraries for disruptions in specific genes provides a reliable, moderate-throughput, reverse screening strategy. This strategy can be applied to those organisms for which there are methods for high efficient transformation, but for which homologous recombination occurs at low frequency. Organisms that would benefit from this approach include algae, plants and other eukaryotes. In this study, we identified 82.5% of the screened insertion sites in Library 1 (~100,000 transformants). Naturally, using a large population of transformants significantly increases the chances of identifying a transformant with an insertion in a specific target gene. However, progressive screens for individual target gene disruptions allow for the termination of the screen once an appropriate insertion into the target gene is identified; this approach could significantly reduce the final number of transformants that would need to be screened. Assuming the use of 4 target primers for a gene of interest and the presence of the transformant of interest within a population of 100,000 transformants, the number of PCR reactions needed to identify a superpool that contains such a transformant would be between 4 and about 416 (depending on whether it is identified in the first or last superpool tested).

For constructing transformant libraries, we recommend the use of a heterologous, plasmid-free, linear marker gene DNA. This type of marker gene DNA allows for the design of stable, highly specific marker primers positioned at the end of the marker gene coding region, which would facilitate the identification of interrupted target genes. Also, the use of a plasmid-free marker gene makes it easier to characterize the genomic regions flanking the inserted DNA using procedures such as RESDA-PCR [27] and TAIL-PCR [28]. Finally, using a plasmid-free marker gene coupled with electroporation-based methodology for transformation of Chlamydomonas results in the generation of transformants with no or very small deletions, which makes it easier to characterize the insertion site and the exact lesion that is generated, which in turn adds to the value of the PCR-based reverse genetics approach.

Unfortunately, the absence of a convenient method to maintain large number of Chlamydomonas transformants requires that the screen be completed with a one-use (without long-term maintenance of transformants) or a cryopreserved pooled library. With the one-use libraries, once an interrupted target gene is identified within the DNA isolated from the pooled population of transformants, the isolation of that specific transformant can be rapid and feasible. In contrast, with the cryopreserved pooled libraries this process can be time consuming and difficult because some transformants may grow slowly or be under-represented within the population. Hence, PCR-based screening of a moderately large number of target genes using a one-use library represent a reliable method for obtaining specific mutants over a relatively short time interval. Cryopreserved pooled libraries would be most advantageous when performing many small independent screens separated in time; it would avoid the generation and arraying of transformants for each screen.

## Materials and methods

### Cell cultures and isolation of transformants

*Chlamydomonas reinhardtii *strain D66 (CC-4425) (*nit2^- ^cw15 mt^+^*) [29] was used as the parental strain for the transformation experiments described in this work. Liquid and solid cultures of this alga were grown in Tris-acetate phosphate (TAP) medium under continuous light (~60 μmol photon m^-2^s^-1^) at 23°C [30]. D66 transformants were generated by the introduction of a 1.7 kb PCR-fragment containing the *AphVIII *gene under the control of the *PSAD *promoter. The transforming fragment was amplified from the pSL72 plasmid [31] using the RIM-F2 (5'-ACCAATCGTCACACGAGC-3') and RIM-R2 (5'-CTTTCCATCGGCCCAGCAAC-3') primers. Transformation was performed by electroporation using a modification of the procedure reported by Shimogawara et al. [32]. Briefly, the cells were collected by centrifugation at 3000 × *g *for 5 min and resuspended in TAP medium supplemented with 40 mM sucrose to a final cell density of 1-4 × 10^8 ^cells/mL. This cell suspension (250 μL) was placed into a disposable 4-mm gap electroporation cuvette (Bio-Rad., Hercules, CA). PCR-amplified marker gene DNA was analyzed by gel electrophoresis in 1% agarose gels. Amplified fragments were excised from the gel and purified using the QIAquick PCR Purification Kit (Qiagen, Valencia, CA, USA). The purified marker gene DNA was then quantified spectrophotometrically using a Nanodrop ND-1000 (Thermo Scientific), and 100 ng of this DNA was added to the electroporation cuvette, which was then incubated at 4°C for 10 min. An exponential electric pulse of 0.8 kV at a capacitance of 25 μF was applied to the sample using the Gene Pulser II (Bio-Rad, Hecules, CA) electroporation apparatus. After electroporation, cells were transferred to 10 mL of fresh liquid TAP medium and incubated for 16-18 h at 23°C under dim light to allow for accumulation of the AphVIII protein. The dim light and the initial shock of the electroporation procedure would reduce the cell divisions that occur during this incubation time, which in turns would minimize the probabilities of obtaining more than one colony representing the same transformant (with exactly the same insertion site). The cells were then collected by centrifugation at 3000 × g for 5 min, resuspended in 1 mL liquid TAP medium, spread onto agar plates containing TAP medium supplemented with 5 μg/mL of paromomycin, and incubated for 7-15 d in the light (~60 μmol photon m^-2^s^-1^). Individual colonies were picked by touching them with the tip of a toothpick, and transferring them to a well of a 96-well microtiter plate; each well contained 200 μL of liquid TAP medium. The plates were then wrapped in parafilm to minimize evaporation. Each of the 96-well microtiter plates is considered a "pool". The 96-well microtiter plates were incubated at 23°C for 4-7 d. At the end of this growth period, aliquots (25-50 μl each) of cells from each well of individual 96-well plates were combined and used to inoculate a 'transformant pool culture' (into 30 mL TAP medium); the cells were then grown until they reached mid logarithmic phase (2-3 d). If necessary, 96-well plates were kept alive for an additional period (1-2 months) by transferring small aliquots (20-50 μL) from each well to new 96-well microtiter plates containing fresh liquid TAP medium (150 μL).

### DNA isolation and PCR procedures

The pooled transformant cultures (from each microtitre plate) were used for genomic DNA isolation; the DNA preparation from each pool culture represents a DNA "pool". Isolation of genomic DNA was performed using a standard phenol-chloroform extraction method as described previously [33]. The DNA prepared from each pool was diluted to 100 ng/μL and equal volumes of 10 different DNA pools (from 10 different microtitre plates) were combined to form a DNA "superpool". PCR reactions using DNA from "superpools" as template were performed in a final volume of 25 μL and contained 0.4 pmoles of each primer, 0.2 mM of each of the dNTPs, 0.2 U of Taq DNA polymerase (Qiagen, Valencia, CA), 2.5 μL of 10 × Qiagen Taq DNA polymerase buffer, 5 μL Q solution (Qiagen, Valencia, CA), 100 ng of DNA template, and distilled water to make up the remainder of the 25 μL volume. The previously designed RB1 (5'-ATGGGGCGGTATCGGAGGAAAAG-3') and RB2 (5'-TACCGGCTGTTGGACGAGTTCTTCTG-3') primers [27] specifically anneal to the *AphVIII *gene and were used as specific marker gene primers (Figure 1). Target gene specific primers were designed largely with PrimerSelect software (DNAstar 7.1.0, Lasergene) using the default setting for hairpin formation and dimer duplexing; primer lengths were between 18 and 26 nucleotides. The PCR conditions used for screening the genomic DNA superpools were: pre-incubation at 95°C for 5 min followed by 35 cycles of sequential denaturation at 95°C for 30 s, annealing at 60°C for 30 s, and amplification at 72°C for 2 min. PCR products were separated by gel electrophoresis in 1% agarose gels. When needed, reamplification of weak bands was performed by inserting a 200 μL pipette tip directly into the desired DNA band that was resolved on the agarose gel. The tip was then agitated in 50 μL of water and 1 μL of this water was used as a template for reamplification [27]. The reamplified product was excised and extracted from the gel by centrifugation as previously described [27], or by using the QIAquick PCR Purification Kit (Qiagen, Valencia, CA, USA); similar results were obtaining with the two methods. For colony PCR we resuspended a 5-10 μL of cell culture in 50 μL of 10 mM EDTA, incubated this solution at 100°C for 5 min, centrifuged the cell suspension at 12000 × g for 1 min, and used 1.5 μL of the supernatant as a template for PCR reactions. Alternatively, we used the FTA kit pk/1 (Whatman, Piscataway, NJ, USA) following the manufacturer's recommendations. Both methods gave similar results.

### Southern-blot analysis

Isolation and enzymatic digestion of genomic DNA, electrophoretic fractionation of the DNA fragments, transfer of the DNA fragments to a nylon membrane, labeling of specific DNA probes, hybridization of the probes to the immobilized fragments and washing of the hybridized membrane were performed as previously described [33,34]. Labeling of the specific *AphVIII *gene probe was with [^32^P]dCTP by PCR amplification of a 360 bp fragment using the PARAU (5'-GAGGATCTGGACGAGGAGCGGAA-3') and PARAL (5'-CCCTCAGAAGAACTCGTCCAACAGC-3') primers.

## Competing interests

The authors declare that they have no competing interests.

## Authors' contributions

DGB is the main intellectual author of this work, has participated in the designing, coordination, construction and screenings of both libraries, collected data from some transformants, and wrote this manuscript. WP has made important intellectual contribution to this work, has participated in the designing, coordination, construction and screening of Library 1, collected data from the sulfate transporters mutants, and participated in the writing of this manuscript. FM, WY, CC, LM, and MP have participated in the construction and screening of Library 1, and collected data from many transformants enlisted in Tables 4 and 5. JJH and AM participated in the construction and screening of Library 2 and collected data from many transformants enlisted in Tables 4 and 5. AG, EF and ARG have participated in the drafting of the manuscript and critically supervised all the approaches used in this work. All authors have read and approved the final manuscript.
